# The regenerating protein 3A: a crucial molecular with dual roles in cancer

**DOI:** 10.1007/s11033-021-06904-x

**Published:** 2021-11-23

**Authors:** Liying Wang, Yanchun Quan, Yanxi Zhu, Xiaoli Xie, Zhiqiang Wang, Long Wang, Xiuhong Wei, Fengyuan Che

**Affiliations:** 1 Department of Clinlical Medicine, Weifang Medical College, Weifang, China; 2 Department of Neurology, Linyi People’s Hospital, Linyi, China; 3 Central Laboratory, Linyi People’s Hospital, Linyi, China; 4 Key Laboratory of Neurophysiology, Linyi People’s Hospital, Linyi, Shandong China; 5 Key Laboratory of Tumor Biology, Linyi People’s Hospital, Linyi, Shandong China; 6 Shandong First Medical University & Shandong Academy of Medical Sciences, Taian, Shandong China

**Keywords:** Regenerating protein REG3A, Gene expression, Receptor, Regulation, Signaling pathway, Cancer

## Abstract

**Introduction:**

REG3A, a member of the third subclass of the Reg family, has been found in a variety of tissues but is not detected in immune cells. In the past decade, it has been determined that REG3A expression is regulated by injury, infection, inflammatory stimuli, and pro-cytokines via different signaling pathways, and it acts as a tissue-repair, bactericidal, and anti-inflammatory molecule in human diseases. Recently, the role of REG3A in cancer has received increasing attention. The present article aims to investigate the structure, expression, regulation, function of REG3A, and to highlight the potential role of REG3A in tumors.

**Methods:**

A detailed literature search and data organization were conducted to find information about the role of REG3A in variety of physiological functions and tumors.

**Results:**

Contradictory roles of REG3A have been reported in different tumor models. Some studies have demonstrated that high expression of REG3A in cancers can be oncogenic. Other studies have shown decreased REG3A expression in cancer cells as well as suppressed tumor growth.

**Conclusions:**

Taken together, better understanding of REG3A may lead to new insights that make it a potentially useful target for cancer therapy.

## Introduction

REG3A (or regenerating islet-derived protein III-alpha) belongs to the REG protein family and is also known as heptocarcinoma-intestine-pancreas (HIP) or pancreatic associated protein (PAP) [1–3]. As a 19-kDa secreted calcium-dependent lectin protein, REG3A has multiple roles in anti-inflammatory, antimicrobial, cell proliferation, cell apoptosis, and tissue repair that have been explored [4–8]. An increasing number of studies have also demonstrated its potential in tumorigenesis and neural development [9, 10]. Here, we review the regulation, function, and potential clinical application of REG3A to identify novel therapeutic targets for cancer treatment.

## Identification and structure

Reg protein was first identified as pancreatic stone protein (PSP), and was observed in pancreatic stones and juice obtained from patients with chronic calcifying pancreatitis in 1979 [11]. As the later studies of Terazono et al. revealed, the protein was named ‘regenerating gene’ (Reg) because of its increased expression in both regenerating and hyperplastic islets [12]. The Reg protein has been found in humans, rats and mice, and homologous proteins exist in hamsters, dogs, pigs, rabbits and other mammals. At present, approximately 17 Reg proteins have been isolated and identified from mammals, and they can be divided into four subfamilies based on their primary protein structure: type I, type II, type III and type IV [13]. Sequences of the members of the four subfamilies are highly conserved because of the homology of amino-acid sequences from the same species [14].

REG3A belongs to the third subclass of the Reg family, which also consists of Reg3β, Reg3γ, and Reg3δ [15]. *REG3A* is located on human chromosome 2p12 and comprises six exons and five introns [16]. Exon 2 contains three mini-exons (2a, 2b, and 2c), indicating the presence of three different transcripts on the alternative use of 5′-exons [17]. REG3A encodes a 19-kDa protein with a full length of 175 amino acids. The entire domain of REG3A contains a signal peptide (1–26aa), propeptide (27–37aa), and C-type lectin-like domain (CLTD; 47–173aa). The signal peptide can be removed in the secretory pathway to form a mature secreted REG3A. Medveczky et al. found that trypsin could cleave the remaining N-terminal of REG3A at the Arg37–Ile38 peptide bond, and the proteolytic processing of REG3A was necessary for peptidoglycan binding [18]. The EPN motif is included in the CLTD and is essential for peptidoglycan binding and bactericidal activity [19].

**Fig. 1:**
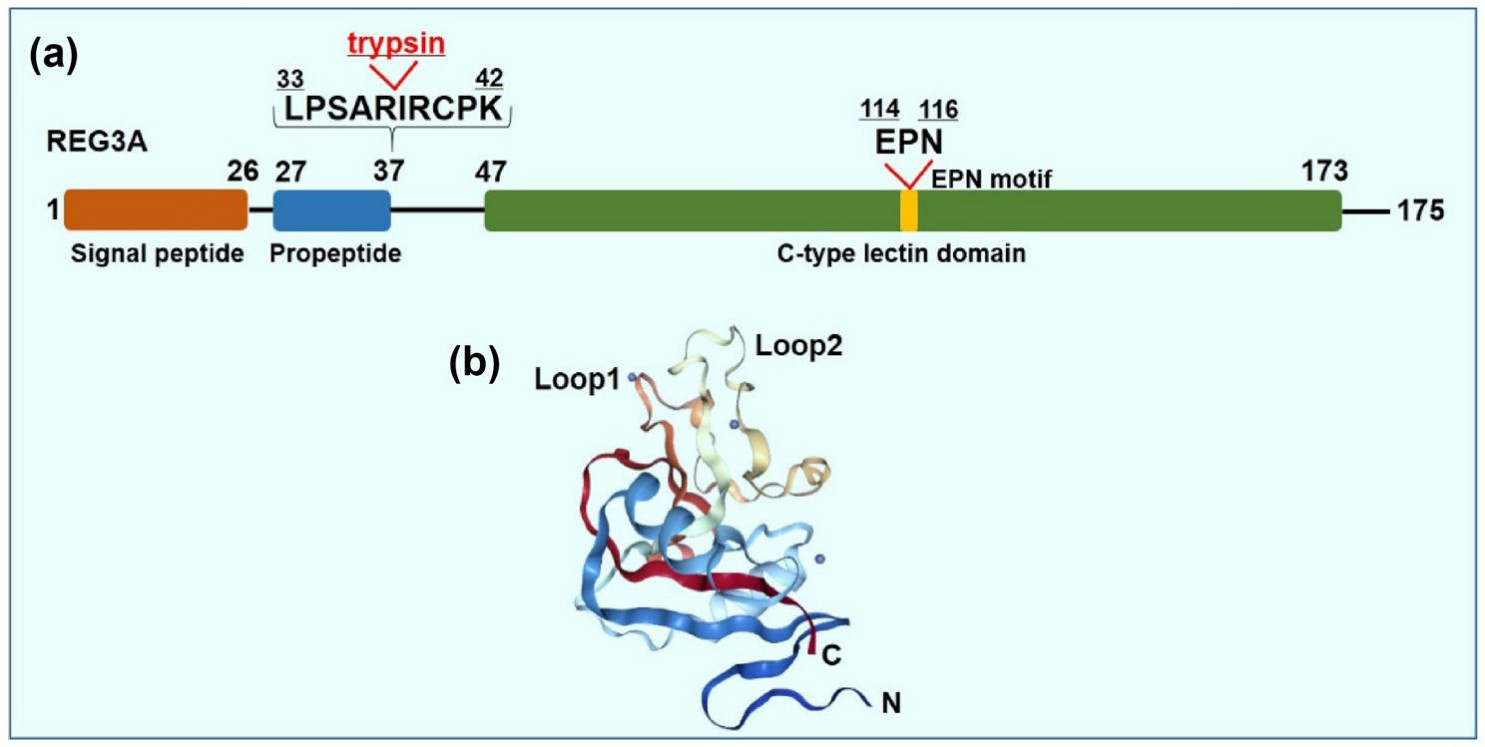
Structure of REG3A. **a** Secondary structure of REG3A. REG3A is a 19-kDa protein that consists of 175 amino acids. REG3A consists of three domains: signal peptide (1–26aa), propeptide (27–37aa) and C-type lectin domain (47–173aa). Trypsin cleavages N-terminal of REG3A at the Arg37–Ile38 peptide bond and the proteolytic processing of REG3A was necessary for peptidoglycan binding. The EPN motif in CTLD domain is essential for bactericidal activity. **b** X-ray structure of REG3A (PDB code 4MTH.pdb), with its characteristic “long loop” structure

The X-ray structure of REG3A obtained from the PDB database is shown in Fig. 1b. The three-dimensional structure of REG3A exhibits 2 long loop regions, β4 strands, and three disulfide bonds (residues 40 ↔ 51, 68 ↔ 171 and 146 ↔ 163). Loop 1 (residues 107–121) and loop 2 (residues 130–145) exhibit two distinct subdomains and the EPN sequence is contained in the loop 1 region [19]. Three disulfide bonds anchored in REG3A impart a high degree of structural rigidity to the protein, which most likely precludes major secondary-structure transitions [20].

## Expression and regulation

### REG3A expression

REG3A has been found in a variety of tissues including the pancreas, small intestine, liver, skin, and gastrointestinal tract, but has not been detected in immune cells [3, 8, 9, 21]. It is constitutively expressed in the intestine, however, REG3A has been detected at low levels in healthy tissues (including the pancreas and skin) and is highly expressed in tissue injury [3, 22]. Recently, emerging evidence has demonstrated the differential expression of REG3A in patients with diabetes, inflammatory bowel disease (IBD), and cancers, which may suggest an important physiological function of REG3A [23–25].

In human IBD colonic mucosa, HIP/PAP (REG3A) mRNA was demonstrated to be overexpressed and may play a role in colon mucosal regeneration [26]. Similarly, *REG3α* gene expression is upregulated in the inflamed gastrointestinal (GI) mucosa and predominantly expressed in the lower GI tract [27]. The homolog of human REG3A in mice, Reg3γ, was found in undifferentiated embryonic stem cells (ESCs) and may be involved in stem cell proliferation [28]. Sakiko et al. also found that Reg3γ was abundantly expressed in epithelial cells in the airway and intestine and showed a gradually increasing pattern along with development [29]. As a regenerating gene, REG3A was abundantly expressed in the lesional skin of psoriasis patients and in IL-17A-induced neonatal human epidermal keratinocytes (NHEKs); however, its expression was very low in differentiated NHEKs [3]. Unlike that in normal human epithelial cells, the expression of REG3A and Reg3γ mRNA was significantly decreased in the skin of diabetic patients and streptozotocin (STZ)-induced experimental type 1 diabetic T1D mice, respectively [5]. Therefore, we suppose thatREG3A has different modes of expression in different diseases.

### REG3A regulation

Many studies have indicated that multiple factors, such as bacteria, injury, or interleukins, could regulate REG3A expression in various pathologies. In the lesional skin of psoriasis patients, skin injury can increase REG3A expression through IL-17/IL-17RA [3]. Furthermore, IL-33 was found to be involved in IL-17-induced REG3A expression; hyperglycemia could inhibit this pathway, leading to decreased REG3A expression in diabetic patients [5]. In hepatocytes, intermittent hypoxia (IH) significantly upregulated HIP/PAP mRNA levels via downregulation of miR-203 levels [30]. Danica et al. observed that gut microbiota-derived peopinate could induce Reg3γ expression via SCFA receptor signaling, and the antibiotic ampicillin (AMT) and rifaximin (RFX) significantly reduced Reg3γ expression [31]. The same conclusion was also drawn in a vancomycin-resistant Enterococcus infection study, wherein antibiotic treatment notably downregulated intestinal Reg3γ expression and the stimulation of TLR4 induced Reg3γ expression [32]. The probiotic Bifidobacterium breve colonized in the intestine effectively induced Reg3γ production via the MyD88-Ticam1 pathway [33]. Moreover, caerulein increased PSP/reg and PAP III expression in the rat exocrine pancreas [34].

IL-22, which regulates host defense at barrier surfaces, induces Reg3γ and Reg3β expression in colonic epithelial cells to enhance early host defense against attaching and effacing (A/E) bacterial infection. STAT3 may participate in the IL-22-Reg3γ pathway [35]. MyD88, an intracellular adaptor in most TLR-mediated signaling pathways, mediates that signals that induce Reg3γ expression; Reg3γ transcription and protein expression were markedly decreased in MyD88-deficient mice [36]. The proinflammatory cytokine IL-8 was also found to dose-dependently induce Reg protein production and Reg promoter activity in cultured ECC10 cells [37]. In addition to the above regulation, human chorionic gonadotropin (hCG) stimulation significantly induced PAP-III mRNA expression at the time of ovulation, which provided evidence that the ovulatory process was comparable to an inflammatory reaction. [38]. Therefore, REG3A may be regulated by many factors in various disease models.

## REG3A signaling

The expression of REG3A could be regulated by multiple signals; however, different signaling pathways could be activated or regulated by REG3A in human disease. Thus, we next discuss the involvement of REG3A in regulatory mechanisms.

### REG3A receptor

In 2000, Kobayashi et al. isolated a Reg-binding protein cDNA from a rat islet cDNA library [39]. The putative receptor cDNA was 2760 bp in length and encoded a 919-aa protein. The homology search revealed that the cDNA and its encoded sequence belonged to the human multiple exostoses (EXT) family and exhibited 97% homology with EXT-like gene 3 (EXTL3). The RNase protection assay detected the expression of Reg receptor mRNA in the liver, kidney, stomach, small intestine, and brain, similar to the expression of other EXT family genes. The broad expression of the Reg receptor suggests that the Reg/Reg receptor signal may be involved in multiple cell types.

In 2012, Lai et al. confirmed EXTL3 as a functional REG3A receptor in keratinocytes [3]. REG3A could bind to EXTL3 on keratinocytes, and silencing of EXTL3 significantly blocked the activity of REG3A. Similarly, the administration of EXTL3 antibody abolished the inhibitory effects of RegIIIγ on differentiation in vivo. However, the details of the interaction between REG3A and the putative receptor EXTL3 are still unclear. In 2016, Lai et al. further evaluated EXTL3 as the receptor for REG3A [5] and found that N-EXTL3 (1–548aa), and full-length EXTL3 was necessary for C-REG3A (CTLD) binding. Silencing of EXTL3 blocked the downstream signal and SHP-1 (Src homology region 2 domain-containing protein tyrosine phosphatase 1) expression induced by REG3A. Therefore, EXTL3 may be a potential receptor for REG3A. However, it is still unknown whether there are other receptors for REG3A in other types of cells or tissues.

### REG3A gene regulatory effects

**Fig. 2:**
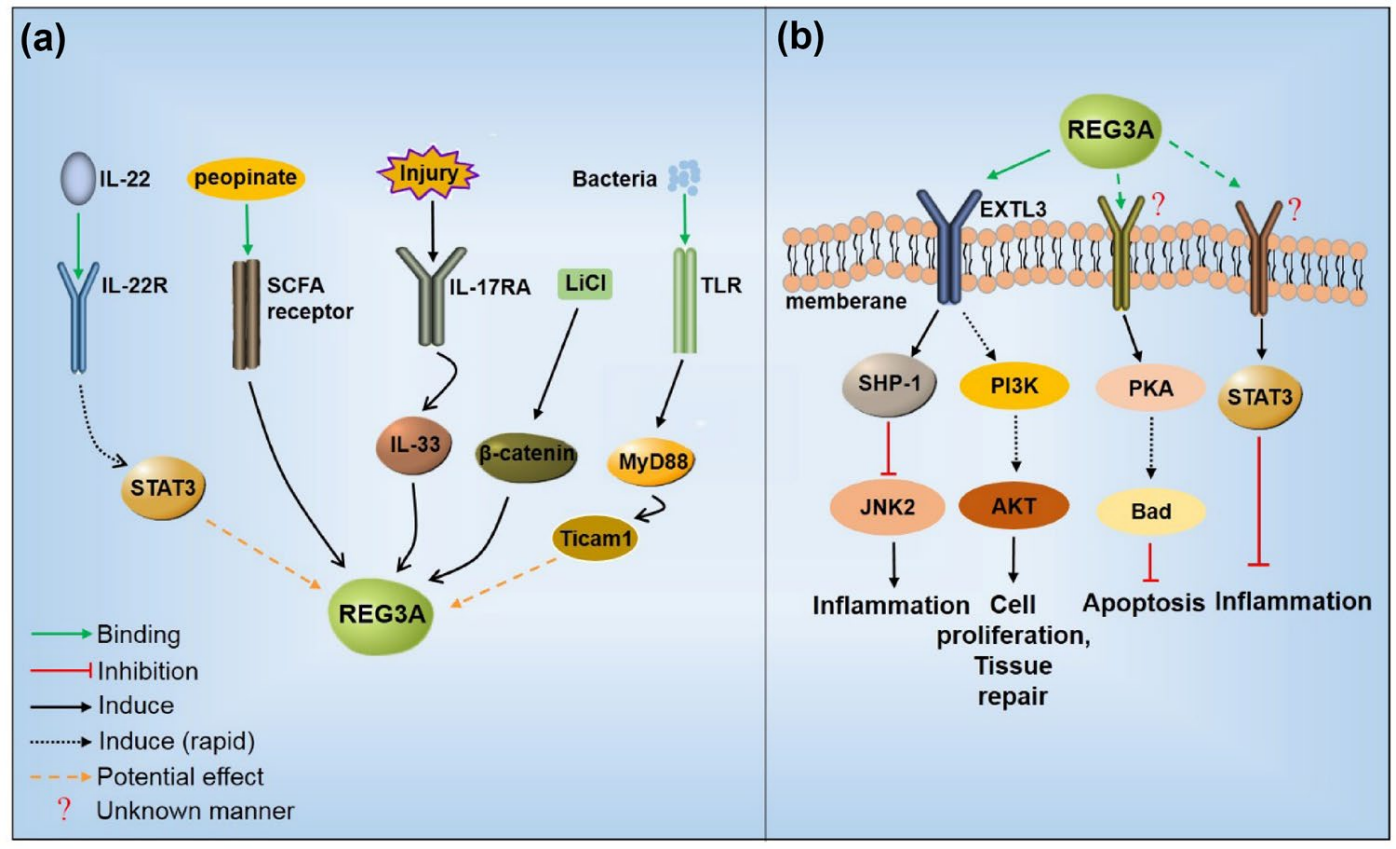
Regulation and signaling of REG3A. **a** REG3A could be regulated by various stimulators. Once our body suffered injury, infection or other stimuli, REG3A expression was induced through the recognition receptor and adaptors, including TLR, IL-17RA, IL-22R, SCFA receptor or MyD88. **b** As a secreted protein, EXTL3 may serve as one of the potential receptors for REG3A according to current research. Other potential receptors have not yet been identified. REG3A could promote cell proliferation, suppress inflammation and apoptosis via regulating multiple signaling pathways

REG3A is secreted into the extracellular space after maturation and participates in multiple signaling pathways (Fig. 2). In hepatoma cells, REG3A mRNA expression could be gradually induced after 72 h of lithium chloride (LiCl) treatment, and β-catenin siRNA effectively inhibited REG3A expression in cells treated with LiCl [40]. Recombinant HIP/PAP induced PKA activation to phosphorylate Bad at Ser-112 after treatment for 30 min to protect hepatocytes against apoptosis induced by TNF-α + Act D (actinomycin-D) [41]. HIP/PAP I also modulated STAT3/SOCS3 and NF-κB pathways after 30 min or 120 min addition to inhibit the inflammatory response in a positive feedback mechanism with a strong induction of HIP/PAP I expression in response to HIP/PAP I itself in epithelial cells [42]. Ingenuity pathway analysis (IPA) software showed that Reg may activate inflammation and cell proliferation and may attenuate cell death through the JAK/STAT, NF-κB, MAPK (ERK1/2, P38, and JNK), PLC, and PI3K/AKT signaling pathways [43]. In keratinocytes, REG3A significantly activated PI3K to induce the phosphorylation of AKT after treatment for 1 h, since EXTL3 shRNA and PI3K inhibitors markedly blocked the activity of REG3A [3]. REG3A inhibits TLR3-dependent phosphor-JNK2 by modulating the negative regulator SHP-1 after skin injury. However, hyperglycemia blocked REG3A-SHP-1 signaling, leading to increased JNK2 phosphorylation and excessive TNF-α and IL-6 levels in diabetic skin wounds after 1 h or 24 h of treatment [5]. The physiological roles of REG3A are constantly being studied, and the signaling pathways involved need to be further explored.

## Multiple roles of REG3A

### REG3A as a tissue repair protein

REG3A was originally discovered as a regenerating gene and has possible roles in the replication, growth, and maturation of islet β-cells [12]. The tissue regeneration role of REG3A has been observed in different tissues in addition to the pancreas. REG3A acts as a paracrine hepatic growth factor that stimulates the proliferation of liver cells, increases liver weight, and accelerates liver regeneration after partial hepatectomy [44]. In chronic gastritis and cultured gastric mucosal cells, the Reg protein stimulates gastric mucosal cell proliferation and increases the number of Reg-positive cells in patients with *Helicobacter pylori*-positive chronic gastritis specimens, suggesting the potential role of the Reg protein in the regeneration of the gastric mucosa [45]. After skin injury, REG3A promotes skin keratinocyte proliferation and wound re-epithelialization during normal wound repair and psoriasis [3]. Therefore, REG3A plays an important role in tissue repair.

### REG3A as an anti-inflammatory cytokine

The anti-inflammatory effects of REG3A have been reported in many studies. Serum PSP/REG levels in acute pancreatitis are significantly higher than those in chronic pancreatitis [46]. In mice with dextran sulfate sodium (DSS)-induced colitis, Reg3γ protein expression was upregulated and the STAT3-associated cytokines (such as IL-6, IL-17, and IL-22)/Reg3γ axis played a pivotal role in the acute phase of colitis [47]. These results suggest the critical effects of PSP/REG on inflammation. Moreover, patients with active IBD have increased serum PAP levels compared to controls, and incubation with PAP inhibits TNF-α-induced NF-κB activation and reduces proinflammatory cytokines in monocytic, epithelial, and endothelial cells [48]. Furthermore, inflammatory responses are known to be necessary and important for wound healing. Lai et al. discovered that REG3A could inhibit TLR3-induced inflammation after skin injury. The inhibitory effect of REG3A on TLR3-induced TNF-α and IL-6 is mediated by the negative regulatory factor SHP-1 [5]. Consequently, the anti-inflammatory factor REG3A plays a pivotal role in inflammatory diseases.

### REG3A as an antimicrobial peptide

The antibacterial activity of REG3A was discovered in 1991. Iovanna et al. found that Escherichia coli aggregated when incubated with PAP (0.5 M) and showed that PAP may control the proliferation of bacteria [49]. Since then, the antibacterial function and mechanism of REG3A against microbial infection have gradually been elucidate. MyD88-deficient mice harbor more intestinal *Listeria monocytogenes*; however, administration of Reg3γ markedly diminished the number of *L. monocytogenes* [36]. In alcoholic steatohepatitis, overexpression of Reg3γ restricted the colonization of mucosa-associated bacteria, reduced bacterial translocation, and reduced ethanol-induced liver damage [50]. Bacterial flagellin induces Reg3γ expression through TLR5 and enhances intestinal defense against vancomycin-resistant Enterococcus infection [51]. The antibacterial Reg3γ forms a physical separation between the host and microbiota in the small intestine and enhances intestinal adaptive immune responses [52]. The molecular basis of Reg3γ/REG3A bactericidal activity was determined; Reg3γ/REG3A binds to its bacterial targets by recognizing the cell-wall peptidoglycan carbohydrate backbone in a calcium-independent manner through the conserved “EPN” motif for bacterial killing [1, 19].

### REG3A in cancer

**Fig. 3:**
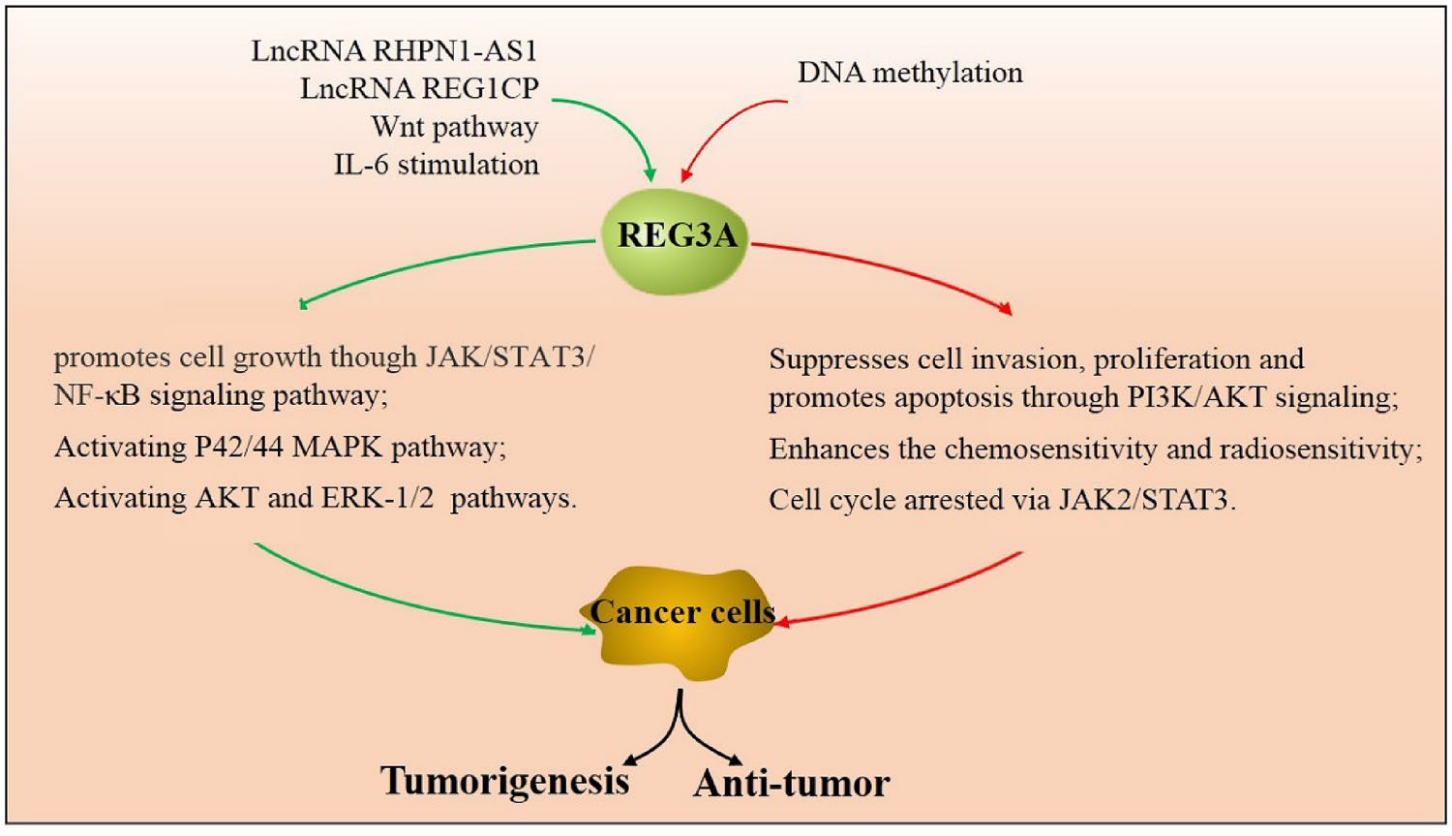
Roles of REG3A in cancer. REG3A has dual roles in cancer research, which is unlike in non-cancer cells. For one side, REG3A was high expressed in many cancer tissues and cell lines and promoted cancer cell proliferation, migration and invasion through activating JAK/STAT3, AKT or ERK signaling pathways. The overexpressed REG3A could be regulated by LncRNA, inflammatory environment, or Wnt pathway. For another, REG3A had low levels in part of tumor tissues. And this expression suppression may be related to DNA methylation. Cells showed growth inhibition, cell cycle arrested and enhanced chemosensitivity and radiosensitivity when REG3A was overexpression. Therefore, REG3A acted as a tumorigenesis or anti-tumor factor in different tumors

Since 1998, REG3A has been discovered to be expressed in various tumor cells and tissues, including in gastric cancer, breast cancer, glioma, pancreatic ductal adenocarcinoma, hepatocellular carcinoma, colorectal carcinoma, and pancreatic cancer (Table 1). Several studies have shown that REG3A is highly expressed in cancer cells and promotes tumorigenesis [40, 53–59, 62, 63, 65]. However, other studies have demonstrated low levels of REG3A expression in cancer cells and a negative correlation with cancer progression (Fig. 3) [60, 61, 64]. Thus, contradictory roles of REG3A in modulating tumor development and progression have been noted in different tumor models.

**Table 1:** Expression and clinical data of REG3A in human tumors

Tumor type	Disease	No. of samples	Methods	Expression level	Association	References
Liver	Hepatocellular carcinomas	42	RNA extraction, Northern blot and RT–PCR	Increased	Correlated to the β-catenin status	[40]
Liver	Hepatocellular carcinomas	192	ELISA	Increased	Serum marker of HCC	[53]
Liver, bile duct	Hepatocarcinoma and Cholangiocarcinoma	35	Northern blot, ELISA, RT-PCR and IHC	Increased	Implicated in differentiation and proliferation	[54]
Liver	Hepatocellular carcinomas	88	RT-PCR and IHC	Increased	REG3A/p42/44 pathway/PDGF-ββ signaling and tumor-stroma crosstalk	[55]
Pancreas	Pancreatic ductal adenocarcinoma	76	RT-PCR and IHC	Increased	Correlated with nodal involvement, distant metastasis and short survival	[56]
Pancreas	Pancreatic cancer	36	PCR and Western blotting	Increased	Coexistence of SOCS3 methylation	[57]
Pancreas	Pancreatic ductal adenocarcinoma	251	ELISA and IHC	Increased	Favors perineural invasion in PDA	[58]
Gastric	Gastric cancer	41	PCR and Western blotting	Increased	Activation of the JAK2/STAT3 signal pathway	[59]
Gastric	Gastric adenocarcinomas	30	RT-PCR and IHC	Decreased	DNA methylation	[60]
Gastric	Gastric cancer	34	PCR and Western blotting	Decreased	PI3K/Akt-GSK3β signaling pathway	[61]
Colorectal	Colorectal carcinoma	331	IHC, TMA and WB	Increased	Reliable markers of favorable prognosis of CRC patients	[62]
Colorectal	Colorectal cancer	82	RT-PCR and IHC	Increased	Activating AKT and ERK1/2 pathways	[63]
Breast	Breast cancer	15	RT-PCR	Decreased	Down-regulated protein of key factors with JAK2/STAT3 pathway	[64]

#### Promoting effect of REG3A in cancer

REG3A has long been studied as a proliferation-promoting factor in several non-tumor cells, as mentioned earlier. Thus, we speculated about the potential relationship between REG3A overexpression and uncontrolled tumor growth. In pancreatic ductal adenocarcinoma, REG3A was overexpressed in 79% (30/38) of pancreatic tissue samples and was correlated with nodal involvement, distant metastasis, and short survival [56]. In patients with pancreatic cancer (PaC), REG3A overexpression coexisted with SOCS3 methylation to promote PaC cell growth through the JAK/STAT3/NF-κB signaling pathway [57]. Furthermore, upregulation of REG3A in colorectal cancer cells was correlated with larger tumor size, poorer differentiation, higher tumor stage, and lower survival rate [63]. In human hepatocellular carcinomas (HCC), tumor-stroma crosstalk enhanced REG3A expression and significantly increased the proliferation of HCC cells via the p42/44 pathway/PDGF-ββ signaling [55]. In glioma cells, REG3A functions as a downstream target gene of lncRNA RHPN1-AS1 and participates in the proliferation, migration, and invasion activity regulated by lncRNA RHPN1-AS1 [66]. In colorectal cancer, REG3A forms an RNA–DNA triplex with lncRNA REG1CP to promote cancer cell-cycle progression and tumorigenicity and is associated with poor patient outcomes [67]. Therefore, the significantly high expression of REG3A implies that it could play an oncogenic role in various cancers.

#### Inhibitory effect of REG3A in cancer

However, some contrary evidence has been observed in recent studies. In primary human gastric cancers, REG3A was found to be downregulated in 67% (20 out of 30) samples and could be restored by the demethylating agent 5-Aza-dC, indicating that the loss of REG3A may be associated with DNA methylation [60]. REG3A expression was also significantly reduced in primary human breast and gastric cancers [61, 64]. Inhibition of proliferation, induction of apoptosis, cell-cycle arrest, and repression of cell invasion and migration were observed after REG3A overexpression in cancer cells through the JAK2/STAT3 or PI3K/Akt signaling pathways [61, 64]. In head and neck squamous cell carcinoma (HNSCC), cells transfected with REG3A exhibited significantly decreased cell proliferation and higher chemosensitivity and/or radiosensitivity; REG3A expression was significantly associated with prolonged survival [68]. Thus, REG3A may act as a suppressive factor in these types of cancer, and the downregulation of REG3A may be useful in the diagnosis of such cancers.

## Discussion

In summary, as a multifunctional molecule, REG3A plays important roles in tissue repair and in anti-inflammatory, antibacterial, and anti-apoptotic mechanisms. In addition, the role of REG3A in cancer is receiving increasing attention, as it is expressed in many tumors. However, there are contradictory reports on the roles played by REG3A in different tumors. From the table, REG3A mainly exhibited differential expression and function in malignant tumors, over-expressed REG3A has been mostly found in the digestive system cancer, including hepatocellular carcinoma, pancreatic cancer, gastric cancer, and colorectal cancer. However, low-expressed REG3A was found in gastric cancer and breast cancer. We could notice that the expression of REG3A in gastric cancer exhibited inconsistent in different research groups. The inconsistent results may be due to the lack of uniform control and further studies are needed to explore the role of REG3A in tumors. In addition, REG3A as a multifunctional protein that may disobey the "one gene-one protein-one function" model. However, more and more proteins are not suitable for this model from the current study. One gene may not correspond to a protein due to various events at the DNA (gene rearrangement) and mRNA (alternative splicing) [69]. And one protein may activate different signaling pathways under different diseases to perform different biological functions [70, 71]. Moreover, we speculate that EXTL3 may not be the only specific receptor for REG3A, and REG3A may activate different signaling pathways through other receptors. The multifunctional of REG3A strongly exemplifies the central role of REG3A plays in a variety of cellular functions. Further clarifying the characterization of REG3A may lead to a better understanding of tumor development and progression and indicate a potential target for clinical treatment.

## Abbreviations

REG3A: Regenerating islet-derived protein III-alpha
HIP: Heptocarcinoma-intestine-pancreas
PAP: Pancreatic associated protein
PSP: Pancreatic stone protein
CTLD: C-type lectin-like domain
IBD: Inflammatory bowel disease
GI: Gastrointestinal
ESCs: Embryonic stem cells
NHEKs: Neonatal human epidermal keratinocytes
STZ: Streptozotocin
AMT: Ampicillin
RFX: Rifaximin
VRE: Vancomycin-resistant *Enterococcus*
A/E: Attaching and effacing
hCG: Human chorionic gonadotropin
EXT: Exostoses
EXTL3: EXT-like gene 3
SHP-1: Src homology region 2 domain-containing protein tyrosine phosphatase 1
LiCl: Lithium chloride
IPA: Ingenuity pathway analysis
PaC: Pancreatic cancer
HCC: Hepatocellular carcinomas
PDGF-ββ: Platelet-derived growth factor ββ
HNSCC: Head and neck squamous cell carcinoma
